# A rare case of cavitary lung cancer complicated with mycotic pneumonia and bullous emphysema: A case report and review of the literature

**DOI:** 10.1097/MD.0000000000008927

**Published:** 2017-11-27

**Authors:** Cun-Tao Lu, Rui-Mei Zhang, Heng Wang, Feng-Wei Kong, Wen-Bin Wu, Long-Bo Gong, Miao Zhang

**Affiliations:** a Department of General Thoracic Surgery, Xuzhou Central Hospital Affiliated to Southeast University; b Department of Respiratory Medicine, Xuzhou Infectious Disease Hospital; c Department of Gastrointestinal Surgery, Xuzhou Central Hospital Affiliated to Southeast University, Xuzhou, China.

**Keywords:** bullous emphysema, cavitary lung cancer, cystic lung cancer, mycotic pneumonia, thin-walled

## Abstract

**Rationale:**

The accurate diagnosis and staging of cavitary lung cancer is challenging but essential for the choice of therapy; therefore, the differential diagnosis of cystic pulmonary lesions needs to be elucidated.

**Patient concerns:**

A patient was admitted with multifocal thin-walled cystic lesions in chest computed tomography.

**Diagnoses:**

The patient had been diagnosed as heterogeneous bullous emphysema pathologically about 3 years ago. His diagnosis turned out to be metastatic cavitary lung cancer complicated with fungal pneumonia this time.

**Interventions:**

The patient underwent lung volume reduction surgery during his first hospitalization. Concurrent systemic chemotherapy and whole brain radiotherapy were administered after the diagnosis of cystic lung cancer.

**Outcomes:**

The patient was lost to follow-up after the chemoradiotherapy.

**Lessons:**

Cavitary lung cancer should always be kept in mind during differential diagnosis of pulmonary cystic lesions. Pathological diagnosis by biopsy and surgery could be considered to avoid delayed treatment of malignancy.

## 1 Introduction

Thin-walled cavitary lesion is characterized by its wall less than 4 mm.^[1]^ The incidence of cavitary primary lung cancer is reported to be 2% to 16%.^[2]^ The cavitary lung cancer with a wall > 4 mm has a higher frequency of vascular and lymphatic invasion and bronchiolar obstruction. The thickness of the cavity wall is an independent prognostic factor.^[3]^ Cavitary adenocarcinoma indicates worse prognosis as compared with the noncavitary counterparts; therefore, cavitary and noncavitary adenocarcinoma should be considered as separate entities.^[4,5]^ Similarly, surgically treated cavitary lung cancer has a worse prognosis than noncavitary cases.^[6]^

A giant bulla should be resected in risk patients older than 50 years because of the high incidence of coexisting cancer.^[7]^ Precise staging and timely treatment are essential for patients with cystic lung cancer. However, it is always difficult to obtain sufficient specimen for pathological staining by percutaneous biopsy. Aggressive surgery of the cystic lesion could be considered for a definite diagnosis. The differential diagnosis of cavitary lesions include bronchogenic carcinoma, lymphoma, abscess, pulmonary infarct, and some congenital lesions. The disseminated cavitary lesions may indicate pulmonary metastatic malignancies.^[8]^

Herein, a case of multifocal cystic lung cancer complicated with mycotic pneumonia and bullous emphysema was presented. Literature regarding the clinical characteristics and treatments of cavitary lung cancer is also reviewed briefly.

## 2 Case presentation

**Figure 1:**
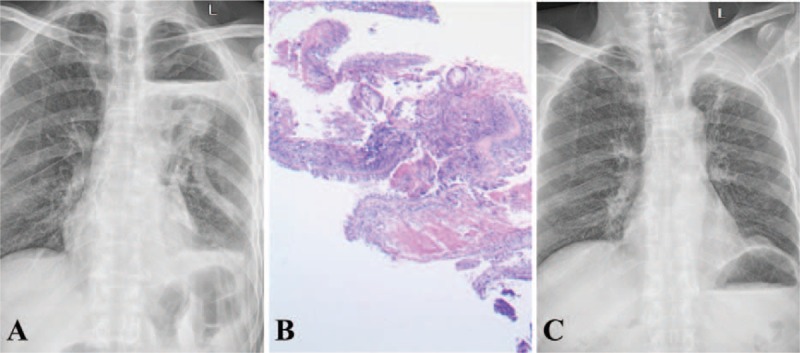
(A) Chest x-ray of the patient indicated pleural effusion 3 days after lung volume reduction surgery on June 27, 2014; (B) Postoperative pathological stain revealed nonspecific inflammation, by H-E staining (×100); (C) Chest x-ray indicated re-expansion of the lobes 3 months after surgery on September 22, 2014.

A 61-year-old male patient was firstly admitted to a local hospital on June 21, 2014, because of chest pain and dyspnea for 3 days, without fever, hemoptysis, hoarseness, or obvious loss of weight. He had a 48 pack-year smoking history, without diabetes mellitus or rheumatism. Serum tumor markers of cytokeratin 19 fragment (CYFRA21–1), carcinoembryonic antigen (CEA), squamous cell carcinoma (SCC), neuron-specific enolase (NSE), alpha fetal protein (AFP), and serum ferritin (SF) were all in normal range. The patient was diagnosed as pneumothorax as the chest X-ray indicated, and the pulmonary lobes were re-expanded after bilateral closed chest drainage (Fig. 1A). Chest computed tomography (CT) was not carried out, although malignancy could not be ruled out definitely. Then, unilateral thoracoscopic lung volume reduction surgery (LVRS) with mediastinal lymph node sampling was performed on June 24, 2014. His postoperative recovery was mainly uneventful except thoracic lymphorrhagia, which was cured after 12 days of fat-free diet. The lymph nodes and resection margins were tumor-negative. The immunohistochemistry staining of the specimen demonstrated positive expression of cytokeratin, epithelial membrane antigen (EMA), vimentin and Ki67 (20%), and negative chromogranin A and synaptophysin. A definite diagnosis was not obtained because the specimen was insufficient for further staining.

**Figure 2:**
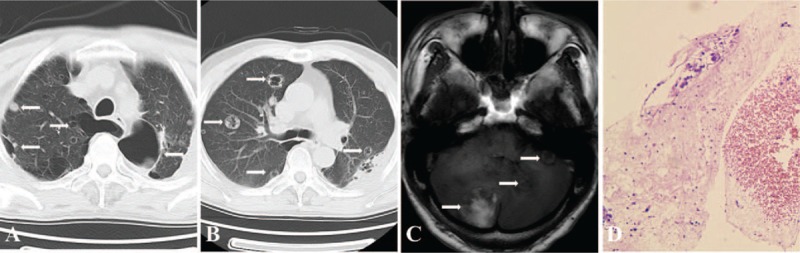
(A, B) Chest CT revealed multifocal thin-walled, cystic lesions (arrow) on April 5, 2017; (C) Cranial MRI showed brain metastasis (arrow); (D) Percutaneous biopsy of the pulmonary cystic lesion demonstrated typical characteristics of poorly differentiated lung adenocarcinoma, by H-E staining (×100).

Nearly 3 years later, the patient was readmitted for dizzy, vomiting, and weight loss for 2 weeks on April 10, 2017. His physical examination showed bilateral coarse breath sounds. Chest CT on admission indicated disseminated cystic pulmonary lesions (Fig. 2A), enlarged mediastinal lymph nodes, and bullous emphysema (Fig. 2B). To rule out fungal pneumonia, tuberculosis, or malignancy, laboratory tests were carried out. Serum tumor marker of CYFRA21–1 was 24.87 ng/mL (0–3.3 ng/mL was considered as normal), while the serum CEA, SCC, NSE, AFP, and SF were all in normal range. Besides, repeated culture of his sputum revealed Candida dubliniensis. Bronchoscopy of this patient showed nothing abnormal. Further cranial magnetic resonance images revealed scattered brain metastasis (Fig. 2C), and the emission CT showed bone metastasis. Positron emission tomography (PET) was not performed because it was not covered by his health insurance.

Then CT-guided biopsy of the cystic lesion in left upper lung was performed, which revealed poorly differentiated pulmonary adenocarcinoma (Fig. 2D). Meanwhile, immunohistochemistry tests of this specimen demonstrated positive expression of cytokeratin 7, EMA and Ki67+(50%), and negative thyroid transcription factor-1 (TTF-1) and anaplastic lymphoma kinase (ALK). No fungal elements were detected microscopically. Further molecular examinations such as epidermal growth factor receptor (EGFR) and ALK was not carried out for financial reasons.

On the basis of these findings, the diagnosis of this patient was established as metastatic cavitary lung cancer complicated with mycotic pneumonia and bullous emphysema. Thus, concurrent chemotherapy and whole brain radiotherapy were decided after multidisciplinary consultation. Subsequently, he received 1 cycle of chemotherapy using gemcitabine (1000 mg/ m^2^ of body surface area) and cisplatin (75 mg/ m^2^ of body surface area), zoledronic acid (4 mg), and whole-brain radiotherapy. Meanwhile, antifungal fluconazole injection (400 mg per day) was administered for 2 weeks. Nevertheless, the patient was lost to follow-up thereafter.

## 3 Discussion

In the era of precision medicine, an accurate diagnosis of cavitary lung cancer complicated with other cystic lesions is sometimes challenging. Accordingly, there are several issues should be elucidated.

First, the formation of cavity in lung cancer is correlated with central necrosis of the lesion, check-valve mechanism, disruption or rupture of the alveolar wall, development in preexisting bullae, cyst or honeycombing, and autophagy of tumor cells.^[9]^

Second, numerous studies have demonstrated the relationship of chronic obstructive pulmonary disease and the development of lung cancer.^[10]^ Both airflow obstruction and emphysema are independent risk factors for lung cancer.^[11]^ Similarly, patients with combined pulmonary fibrosis, emphysema, and idiopathic pulmonary fibrosis have a higher risk of lung cancer than those with merely emphysema.^[12]^ Primary lung cancer and secondary pulmonary metastasis seem to be triggering factors for subacute invasive pulmonary aspergillosis, which is correlated with the development of cavities.^[13]^ In addition, pulmonary infection is associated with lung cancer.^[14]^ Therefore, effective infection control might have a positive effect on the burden of infection-associated cancer.^[15]^

Third, the lobulation sign, spiculation sign, and notched margins on the outer wall are typical characteristics of cancer cavity.^[16]^ The differential diagnosis of cystic lung diseases include lymphangioleiomyomatosis, Birt–Hogg–Dubé syndrome, congenital cystic lung disease, and pneumonia.^[17]^ Synchronous fungal infection and lung cancer with brain metastasis should also be considered,^[18]^ although the coexistence of fungal infection and lung cancer is relatively uncommon.^[19]^ Biopsy or surgical resection could be considered during the differential diagnosis of cystic lung lesions.

Low-dose CT is an early detection approach for lung cancer. If CT and PET images demonstrate thin-walled cavities with uneven thicken wall and increased standard uptake value, lung cancer should be considered.^[20]^ However, progressive wall thickening or appearance of a nodule in or out of a cyst raises the suspicion of lung cancer.^[21]^ If a cavitary tumor shows irregularity, notching, inhomogeneous thickening of the wall, or an enlarging tendency during the follow-up, surgical resection for a definite diagnosis is necessary.^[9]^ Percutaneous needle washing is a useful diagnostic procedure for pulmonary thin-walled cavitary lesions.^[1]^ Besides, dynamic contrast enhanced MRI is helpful to determine the biopsy site of cavitary lung lesions.^[22]^ Pulmonary mycobacterial disease mimics lung cancer, therefore biopsy is essential to avoid unnecessary surgery.^[23]^

Nevertheless, it may be challenging to obtain sufficient specimen from thin-walled cavitary lesions. Circulating tumor cells (CTCs) in peripheral blood could be utilized as tumor markers.^[24]^ Circulating cell-free DNA harbors genetic and epigenetic characteristics of the original tumor, which might be used to identify hidden carcinoma, especially when the biopsy is unavailable or insufficient.^[25]^ However, the detection of lung cancer using CTCs is very challenging for its rarity.^[26]^

It is noteworthy that periodic CT should be taken for patients with suspected cystic malignancy, with the aim to diminish misdiagnosis and delayed surgery. Meanwhile, close follow-up after resection of cystic lesions is also necessary, because patients without definite diagnosis may take the risk of tumor progression. As for this case, incomplete resection of the diseased lung and postoperative lymphatic leakage might contribute to the distal metastasis of cavitary lung cancer.

## 4 Conclusion

The differential diagnosis of thin-walled cavitary lung lesions should always be kept in mind. Biopsy or resection of the lesion could be considered to avoid misdiagnosis, and close follow-up is necessary.
